# Rituximab treatment in Chinese patients with primary angiitis of the central nervous system

**DOI:** 10.3389/fneur.2025.1554989

**Published:** 2025-03-25

**Authors:** Yu-Zhen Wei, Hua-Bing Wang, Lin-Lin Yin, Ai Guo, Lu-lin Zhang, Jia-Li Sun, Ping Lu, Xing-Hu Zhang, De-Cai Tian

**Affiliations:** ^1^Department of Neurology, Beijing Tiantan Hospital, Capital Medical University, Beijing, China; ^2^China National Clinical Research Center for Neurological Diseases, Beijing, China

**Keywords:** primary angiitis of the central nervous system, vasculitis, rituximab, PACNS, PCNSV

## Abstract

**Objectives:**

To assess the efficacy and safety of rituximab (RTX) in Chinese patients with primary angiitis of the central nervous system (PACNS).

**Methods:**

Herein, we present the outcomes of 8 patients who received RTX for PACNS. Seven patients underwent a brain biopsy showing vasculitis, while the remaining patient had a digital subtraction angiogram and clinical findings highly suggestive of vasculitis. Clinical evaluation, laboratory tests, and imaging modalities were performed during the initial RTX administration and follow-up. The Expanded Disability Status Scale (EDSS) disability score was used to assess treatment response and degree of disability.

**Results:**

The median age at diagnosis of the 8 patients (2 females) was 37.0 years. All patients had active disease when RTX treatment was initiated. Five of the eight patients had refractory disease, and received one or more conventional immunosuppressants (IS). Three patients had contraindications or refused conventional IS. Patients were followed up until their death or the final follow-up visit (median 18 months; range: 0–40 months). The median EDSS score at the last visit (median 3.0; range 0–9.5) was lower than before RTX administration (median 6.5; range 1.5–9.5). In 6 patients, RTX administration was associated with a marked reduction in the number of flare-ups. Two of the six patients developed infections: one with pneumonia, and the other with tuberculosis. In one patient, parenchymal gadolinium enhancement persisted, and a new lesion was found following three courses of RTX.

**Conclusion:**

Our data suggest that RTX therapy may be an additional treatment option for Chinese patients with PACNS.

## Introduction

Primary angiitis of the central nervous system (PACNS) is an uncommon form of vasculitis restricted to the brain and spinal cord (1–3). Glucocorticoids (GCs) alone or in combination with traditional immunosuppressive agents (predominantly cyclophosphamide [CYC]), have previously been used to treat this vasculitis (4–6). However, one-quarter of patients fail to respond to treatment, ultimately relapsing (2, 7). Rituximab (RTX), an anti-CD20 monoclonal antibody, is a promising therapeutic option that has been shown to be effective against PACNS (8–10). However, due to the rarity of the disease and the invasive nature of diagnostic methods (biopsy or cerebral angiography), the experience with the use of RTX for the treatment of PACNS with a definitive diagnosis still comes from case reports. Not only that, but these experiences mostly come from the Caucasian populations (11). As far as we know, there is still a lack of reports on the application of RTX in Chinese patients. Herein, we report the findings in eight Chinese patients with PACNS who were treated with RTX.

## Patients and methods

**Figure 1:**
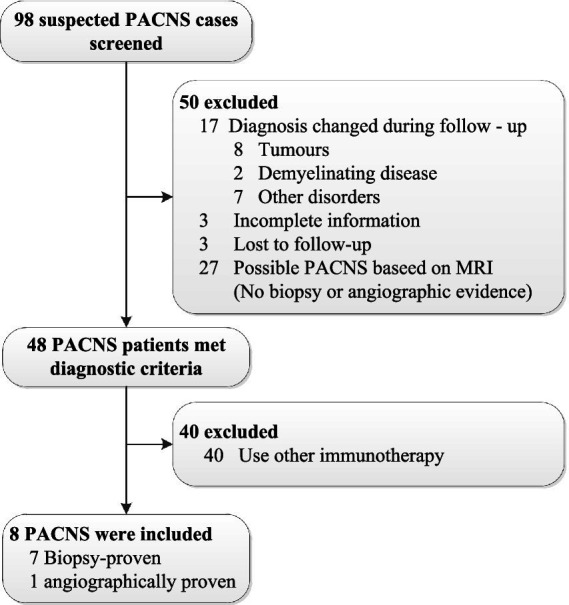
Patient screening and selection for our study.

From January 2018 to March 2022, a total of 98 patients were diagnosed with PANCS in Beijing Tiantan Hospital. After retrospective chart review and follow-up, we finally included 8 patients with PACNS who met the diagnostic criteria according to the Calabrese and Mallek criteria (12) and had applied RTX therapy (Figure 1). Among these 8 patients, 7 patients were found to have vasculitis on brain biopsy, while the remaining patient had a cerebral angiogram and clinical findings highly suggestive of vasculitis.

Clinical evaluation, laboratory tests including erythrocyte sedimentation rate (ESR) levels, autoimmune antibody tests, cerebrospinal fluid routine, biochemistry, etiology, and cytology examinations, as well as imaging modalities, were performed both before and during follow-up. All patients underwent a complete neurological examination performed by a neurologist at the time of diagnosis, at RTX administration, and at subsequent visits. The degree of disability was categorized using the Expanded Disability Status Scale (EDSS) disability score (13) and the modified Rankin scale (mRS) (14). The results of brain magnetic resonance imaging (MRI), conventional digital subtraction angiography (DSA), and magnetic resonance angiography (MRA), were reviewed by a neuroradiologist. Cerebral biopsy specimens were reviewed by a pathologist blinded to the clinical information.

RTX was administered as follows: four patients received two infusions of 500 mg or 1 g RTX at 2-week intervals, while one infusion was repeated every 6 months thereafter (patients 1, 3, 4, and 8). Three patients received 3–4 monthly infusions of RTX as induction therapy during the course of their disease (Patients 2, 5, and 7). One patient received only one cycle of 1 g RTX therapy (patient 6).

Relapse was defined as the recurrence or worsening of PACNS symptoms, or any evidence of worsening of existing lesions and/or new lesions on repeat MRI examinations while the patient was receiving no medication, or a stable dosage of medication (2).

This study was approved by the Ethics Committee of Beijing Tiantan Hospital Affiliated to Capital Medical University, Beijing, People’s Republic of China, and written informed consent was obtained from all participants.

## Results

### Patients’ characteristics

The median age of the 8 patients (2 females) at diagnosis was 37.0 years (range 17–72 years). The median disease duration at the onset of RTX therapy was 15 months (range 3–108 months). The overall characteristics of the cohort are presented in Table 1. The characteristics of each patient are listed in Supplementary Table 1.

**Table 1:** Characteristics of the patients with primary angiitis of the central nervous system (PACNS).

Clinical characteristics	PACNS patients (*n* = 8)
Sex ratio (F/M)	2/6
Age of onset, median (range), years	37 (17–72)
Disease duration before RTX, median (range), months	15 (3–108)
Clinical symptoms, *n* (%)	
Limb weakness	6 (75.0%)
Seizures	4 (50.0%)
Headache	3 (37.5%)
Impaired vigilance	2 (25.0%)
Cognitive impairment	2 (25.0%)
Visual symptoms	1 (12.5%)
Abnormal CSF analysis^a^, *n* (%)	3 (37.5%)
Initial MRI findings	
Multiple lesions	6 (75.0%)
Brain infarcts	3 (37.5%)
Gd-enhanced lesions (intracranial or meningeal)	8 (100.0%)
Parenchymal hemorrhage	6 (75.0%)
Subarachnoid hemorrhage	1 (12.5%)
Vascular stenosis, *n* (%)	1 (12.5%)
Initial treatment, *n* (%)	
Steroids only	3 (37.5%)
Steroids + CYC	3 (37.5%)
Steroids + CsA → CYC	1 (12.5%)
Steroids + CYC → CsA → MMF	1 (12.5%)

a Abnormal CSF analysis: protein > 45 mg/dL or white cell count > 5 cells mm^3^; F, female; M, male; RTX, rituximab; CSF, cerebrospinal fluid; MRI, magnetic resonance imaging; CYC, cyclophosphamide; MMF, mycophenolate mofetil; CsA, cyclosporine A.

The most common symptom at presentation was limb weakness (6 patients), followed by seizures (4 patients). Cerebrospinal fluid (CSF) examination was abnormal (protein >45 mg/dL or white cell count >5 cells mm^3^) in 3 patients. Immunocytochemical analysis was negative for all eight patients. Of the eight patients, six had multiple lesions and two had single lesions. Five patients showed a high signal intensity on diffusion-weighted imaging (DWI). Microbleeds were identified using susceptibility-weighted imaging (SWI) in 6 patients. Subarachnoid hemorrhage was found in one patient (Patient 8). All 8 patients had gadolinium-enhanced lesions. Seven patients who underwent a cerebral stereotactic biopsy presented with transmural cell infiltration: 2 were lymphocytic, 2 were granulomatous, 1 was necrotizing, and lymphocytic infiltrates and necrosis were seen in 2 patients. The interval from disease onset to biopsy was 6 months (range 1–107 months). The pathological results of three patients have been reported in our previous studies (15). Vascular imaging was performed in all eight patients. Patients 1–7 showed no evidence of stenosis and were considered to have small vessel variant. In Patient 8, DSA showed occlusion of the lower trunk of the middle cerebral artery, while high-resolution MRI showed concentric thickening and enhancement of the vessel wall. Therefore, he was considered to be large-medium vessel variant.

### Results of treatment and outcome

The primary results of our study are summarized in Table 1. Prior to RTX therapy, 5 of the 8 patients had refractory disease, and had received one or more conventional immunosuppressants (IS): CYC (5), cyclosporine A (CsA) (2), and mycophenolate mofetil (MMF) (1). Three patients received RTX as remission induction therapy because they had contraindications (CYC allergy: *n* = 1) or refused to accept conventional IS (*n* = 2).

At the initiation of RTX, all patients had active disease based on the presence of PACNS symptoms, the worsening of existing lesions, and/or the appearance of new lesions on MRI/MRA examination. All patients received intravenous pulse methylprednisolone (IVMP) therapy (1 g/day for 3–5 days), followed by a weaning course of oral prednisolone over 3–6 months, in addition to RTX.

Before administration of RTX, all patients underwent screenings for infections, tumors, and immunological status, including hepatitis, HIV and syphilis serology, routine blood and urine test, interferon gamma release assay, chest CT, and abdominal ultrasound, lymphocyte subsets and quantitative measurements of immunoglobulins.

Patients were followed up until their death or the final follow-up visit (median follow-up duration, 18 months; range: 0–40 months).

**Figure 2:**
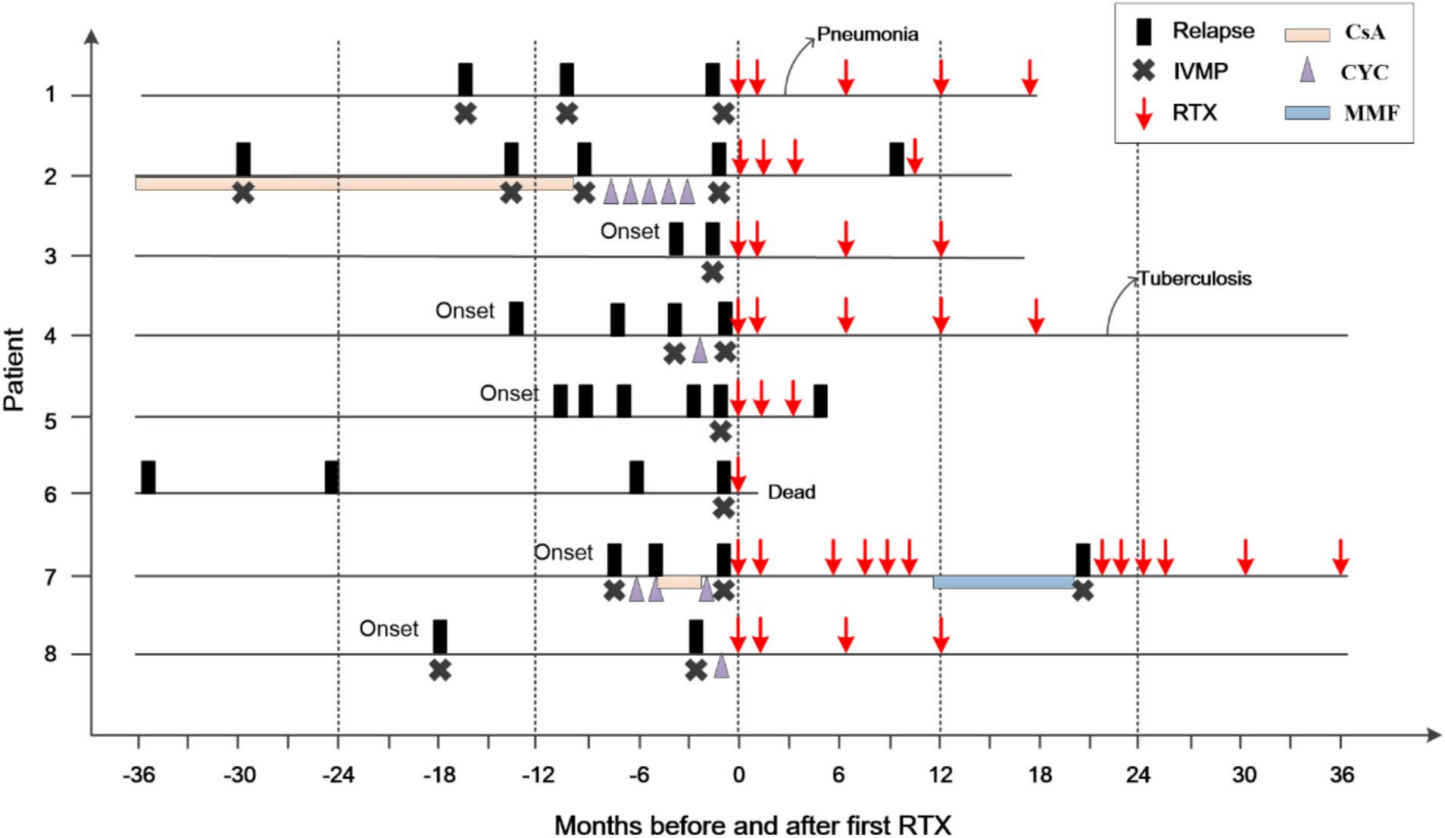
Clinical course of the patients before and after RTX treatment. The zero on the x-axis represents the first administration of rituximab (RTX); IVMP, intravenous methylprednisolone; CsA, cyclosporine; CYC, cyclophosphamide; MMF, mycophenolate mofetil.

In six patients, RTX administration was associated with a marked reduction in the number of flares (patient 1–4, 7, & 8; Supplementary Table 1; Figure 2). Patients 7 and 8 were in critical condition, continued to progress, and did not respond to conventional treatment. After RTX, the lesions in patient 7 did not expand again, and the enhancement disappeared. RTX was switched to MMF after 1 year. However, the size of the lesion subsequently increased, and enhancement recurred. Rituximab was administered again, and the enhancement disappeared 3 months later. Unfortunately, the patient’s neurological function was severely impaired, and the EDSS scores remained high. In Patient 8, the size of the enhancing lesion was reduced, and the EDSS score decreased after RTX.

Two of the 6 patients developed infections following the depletion of B cells, with one developing pneumonia after the second course of RTX (Patient 1). Her condition improved following antibiotic administration, without affecting subsequent treatment. Another patient (patient 4) had a pulmonary nodule after the fifth course of RTX and was diagnosed with tuberculosis. The patient subsequently discontinued RTX treatment. One year after antituberculous treatment, his pulmonary nodule remained, for which he underwent resection. Pathological examination confirmed the diagnosis of tuberculosis. At the last visit (22 months after the fifth RTX treatment), the patient was stable and did not use any other immunosuppressants.

One patient’s symptoms (patient 5) did not improve after three courses of RTX, although his B cells were depleted. At the final follow-up visit 1 month after the third course of RTX, parenchymal gadolinium enhancement persisted, and a new lesion was identified. One patient (patient 6) died at home 10 days after receiving RTX 1 g. As he had serious hemiplegia and difficulty breathing after getting out of bed, the cause of death was suspected to be a pulmonary embolism.

**Figure 3:**
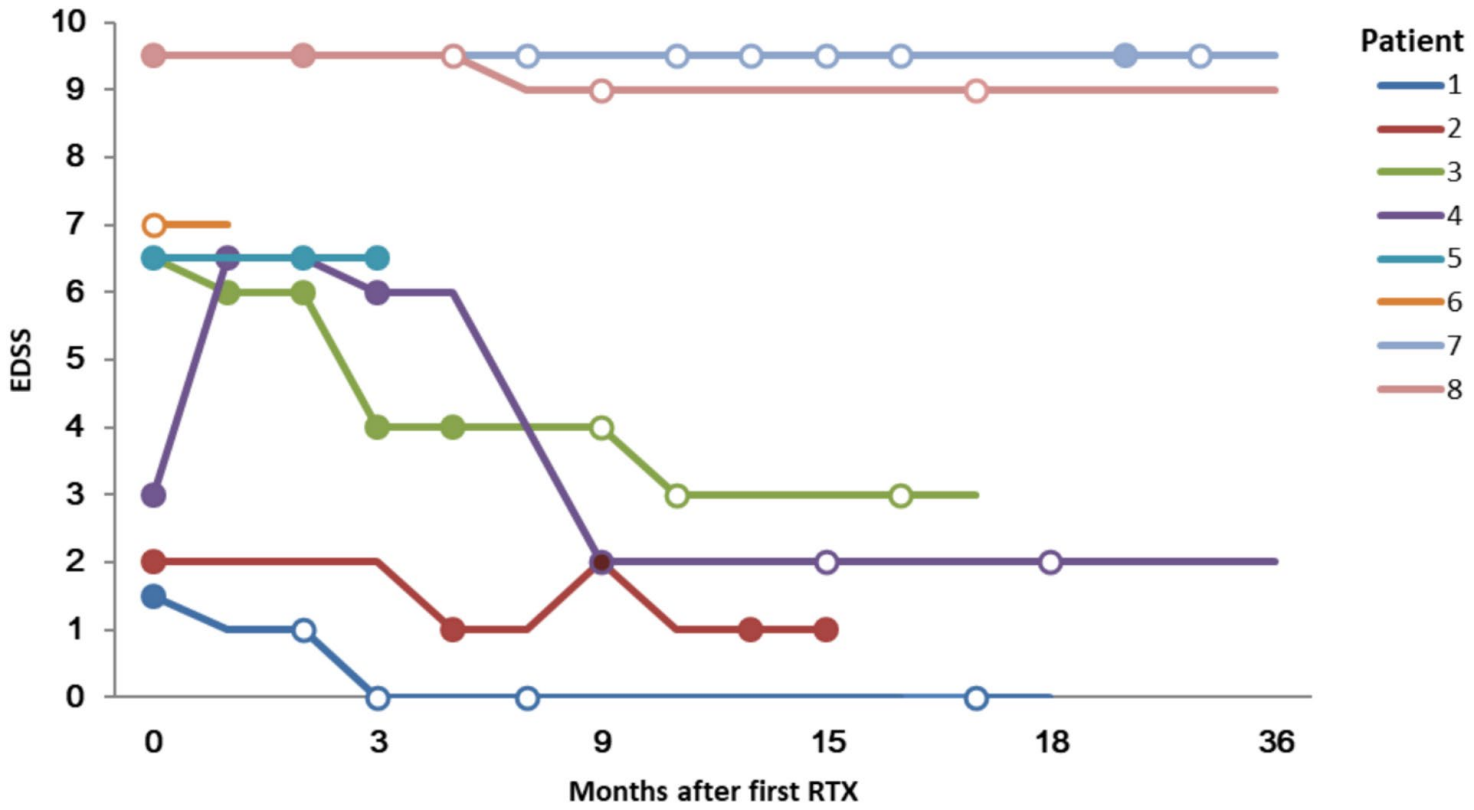
Expanded Disability Status Scale (EDSS) score and lesion enhancement following Rituximab treatment. Solid circles indicate that a gadolinium enhancing lesion was founded; hollow circles indicate no enhancement.

Except for patient 6, the median EDSS score of the remaining 7 patients at the last visit was 3.0 (range 0–9.5). This was lower than prior to RTX administration (median 6.5; range 1.5–9.5) (Figure 3). The mRS scores at the last follow-up (median 4, range 0–5) did not show a significant decrease compared to those before RTX administration (median 4, range 1–5).

## Discussion

PACNS is an inflammatory life-threatening disease associated with a significant risk of morbidity. Relapses can occur in 30–50% of patients with PACNS, thereby increasing the risk of progressive neurological deterioration with severe disability (2, 4, 7). The treatment strategy for primary CNS angiitis is currently a matter of debate, as no randomized controlled trials have yet investigated this rare disorder. The treatment followed for this debilitating disease is largely derived from retrospective/ambispective studies from the Mayo Clinic, French, Cleveland Clinic, German and Indian cohorts (2, 4, 7, 11, 16–18). Glucocorticoids and cyclophosphamide are the current first-line therapies for this condition, although a select group of patients respond poorly to this regimen (2, 7). Whether other combinations or sequences can achieve better results remains to be determined. The expert consensus on the diagnosis and treatment of PACNS in China also recommends glucocorticoids and cyclophosphamide as the first-line treatment (19). For patients either refractory or intolerant to cyclophosphamide, RTX can be considered.

Rituximab, a chimeric monoclonal antibody directed against the B-lymphocyte cell-surface protein CD20 mediates the destruction of B-lymphocytes through a variety of mechanisms, including antibody-dependent cellular cytotoxicity, complement-dependent cytotoxicity, and apoptosis. It has been used successfully for the treatment of systemic vasculitides (20, 21) and in several CNS inflammatory conditions (22). The presence of B lymphocytes in the inflammatory infiltrate supports the use of RTX in PACNS (3, 23). However, only a few case reports regarding the treatment of PACNS with RTX have thus far been published. In these studies, RTX was shown to be an effective alternative to cyclophosphamide in patients with refractory PACNS, and was also effective as a primary treatment after glucocorticoid therapy (8–10, 24). Additional studies are required to evaluate the use of RTX in the treatment of PACNS. To the best of our knowledge, no data on Chinese patients have yet been published, and our study is the first to report on the use of RTX in Chinese patients with PACNS. In our study, RTX induced remission with improvement in neurological status or MRI findings in 6/8 patients. Four of the 6 patients received one or more conventional immunosuppressants. This finding indicates that RTX therapy may be an additional treatment option for Chinese patients with PACNS.

The side effects of RTX include reactions to RTX infusion, systemic infections, hypogammaglobulinemia, and malignancies (25, 26). In published cases, no side effects have been reported during RTX treatment in PACNS patients (10). However, in our study, two of the 6 patients developed infections following B cell depletion (patient 1 and 4). Much of the experience in treating vasculitis with RTX comes from its use in ANCA-associated vasculitis (AAV); with studies of AAV showing that 7, 18, and 12% of the patients enrolled in RCTs experienced severe infections 6, 12, and 18 months after a single RTX cycle, respectively (21, 26–28). However, the results of RCTs and subsequent studies have not shown an alarming frequency of these infections. The RAVE trial analysis, with a predefined combination of the most clinically relevant AEs, showed higher overall rates for the CYC group (33%) than for the RTX group (22%, *p* = 0.01) (21). Currently, CYC is the first-line treatment for PACNS. Further studies are required to compare the safety and efficacy of RTX with those of CYC for the treatment of PACNS. To prevent the reactivation of latent infections or the occurrence of new infections, it is important to monitor infection markers in patients before and during the administration of RTX. Individuals who are prone to infections should receive vaccinations in advance and take precautions to prevent pneumocystis pneumonia.

PACNS is an extremely rare and challenging condition to diagnose and treat. The signs and symptoms are non-specific, and cannot be used to discriminate it from other diseases (29); as such, misdiagnosis is common (30). Cerebral angiography and biopsy are necessary for the definitive diagnosis of PACNS (30). Biopsy remains the gold standard for diagnostic tests. If the disease is not confirmed by biopsy, it is likely to be overdiagnosed (31). Seven of the eight patients in our study were diagnosed using pathology, and the other patient was diagnosed using DSA which ensured the accuracy of the diagnosis.

The Modified Rankin Scale (mRS) is typically used to monitor vascular disease progression. The EDSS is a scale used to assess the degree of disability in patients with demyelinating disease, such as multiple sclerosis. The EDSS quantifies the level of disability by assessing functional impairments in eight functional systems of the central nervous system: Pyramidal, Cerebellar, Brain Stem, Sensory, Bowel & Bladder, Visual, Cerebral, and Other. Higher scores indicate more severe disability (13). In this study, we employed two different scoring systems simultaneously, which helped us to better monitor disease progression and evaluate treatment outcomes.

Although Salvarani et al. considered intracranial hemorrhages infrequent in adults with PACNS (12% of the updated 131 patients) (1). With the application of MRI with gradient-echo T2* and SWI sequences, the detection rate of hemorrhage in patients with PACNS has increased. It accounted for one-third in the French cohort (5) and 100% in the Indian cohort (32). In our previous study, 12/21 patients had microhemorrhages (15). In this study, microbleeds were found in 6/8 patients. Patients with multiple microhemorrhages detected by MRI need to be differentiated from those with cerebral amyloid angiopathy (CAA). In our study, PACNS patients with microhemorrhages had a younger age of onset, which may have aided in the differential diagnosis.

The limitations of this study include the small number of enrolled patients, originating from a single center, the large heterogeneity of patients in terms of disease duration, prior therapies, treatment protocols, and disease severity, lack of controls, and short follow-up duration. However, the rarity of PACNS makes it impossible to conduct randomized controlled trials. Further larger-scale, multicentre studies with standardized rituximab treatment protocols and long-term follow-ups are needed to evaluate the safety and efficacy of RTX compared with conventional treatment.

## Conclusion

Based on our results, we suggest that RTX may be an effective alternative for Chinese patients with PACNS with progressive disease who have previously received or have a contraindication for conventional immunosuppressants. Therefore, the side effects of RTX should be considered when treating this condition. Additional studies are needed to evaluate the safety and efficacy of RTX for the treatment of PACNS.

## Data Availability

The original contributions presented in the study are included in the article/Supplementary material, further inquiries can be directed to the corresponding author.
