# Supplementary material for: Rituximab treatment in Chinese patients with primary angiitis of the central nervous system
Source: Front Neurol. 2025 Mar 25;16:1554989. doi: 10.3389/fneur.2025.1554989 (PMC11977535; doi:10.3389/fneur.2025.1554989)
Supplement: Supplementary file 1 [file Table_1.docx]

Supplementary Table 1. Characteristics of primary angiitis of the central nervous system in patients treated with rituximab.

| Case | 1 | 2 | 3 | 4 | 5 | 6 | 7 | 8 |
| --- | --- | --- | --- | --- | --- | --- | --- | --- |
| Age at RTX initiation /Sex | 27/F | 35/F | 40/M | 39/M | 47/M | 72/M | 17/M | 32/M |
| Stroke risk factors | No | No | Diabetes | Hyperlipidaemia | Hypertension | Diabetes | No | Hypertension |
| Disease duration, before RTX | 108m | 72m | 3m | 12m | 10m | 36m | 8m | 18m |
| Diagnosis way | Biopsy | Biopsy | Biopsy | Biopsy | Biopsy | Biopsy | Biopsy | Angiography |
| Pathology pattern, n (%) | Necrotizing pattern | Granulomatous pattern | Lymphocytic infiltrates and necrosis | Lymphocytic pattern | Lymphocytic pattern | Granulomatous pattern | lymphocytic infiltrates and necrosis | - |
| Previous therapy | IVMP, PDN | IVMP, PDN, CYC, CsA | IVMP, PDN | IVMP, PDN, CYC | IVMP, PDN, CYC | IVMP, PDN | IVMP, PDN, CYC, CsA, MMF | IVMP, PDN, CYC |
| Clinical findings at RTX initiation | Headache,  vertigo, gait imbalance | Headache, seizures, numbness and weakness of the right upper limb | Numbness and weakness of the left limbs | Seizures, motor aphasia,  psychiatric disorders | Headache,  Cognitive dysfunction, visual field defect, weakness of the lower limbs and left upper limbs | Seizures,  left hemiparesis, urine retention | Aphasia,  seizures,  cognitive dysfunction,  drowsiness,  partial blindness, limbs weakness | Seizures, confusion,  dysphagia, left hemiparesis |
| MRI findings at initiation of RTX | Increased extension of the enhancing lesion in both cerebral hemisphere and cerebellum. Susceptibility-weighted imaging showed cerebral microhaemorrhages | New enhancing left parietal lobe lesion with vasogenic oedema and microhaemorrhages | Right frontal lobe  lesion with enhancement and microhaemorrhage | Increased extension of the enhancing lesions in both cerebral hemispheres | Multifocal enhancing lesions | Increase in size of the enhancing lesion | Increased extension of the multifocal lesions with vasogenic oedema and microhaemorrhages | Multifocal enhancing lesions in both cerebral hemisphere s with microhaemorrhages |
| Vascular imaging | No evidence of vessel involvement on the MRA. | No evidence of vessel involvement on the MRA. | No evidence of vessel involvement on the MRA. | No evidence of vessel involvement on the CTA. | No evidence of vessel involvement on the MRA. | No evidence of vessel involvement on the MRA | No evidence of vessel involvement on the MRA. | DSA showed occlusion of the lower trunk of the middle cerebral artery, while high-resolution MRI showed concentric thickening and enhancement of the vessel wall. |
| RTX dosage, g | 0.5g | 1g | 0.5g | 1g | 0.5g | 1g | 0.5g | 0.5g |
| Treatment intervals | 2w-6m | 1m-6m | 2w-6m | 2w-6m | 1m-6m | - | 1m-6m | 2w-6m |
| RTX courses | 5 | 4 | 4 | 5 | 3 | 1 | 12 | 4 |
| No. of attacks before RTX initiation | 4 | 6 | 2 | 4 | 5 | 4 | progressive | 2 |
| No. of attacks during RTX therapy (time after RTX initiation) | 0 | 1 | 0 | 0 | 1 | 0 | 0 | 0 |
| Response (MRI) | Reduction in the lesion size and enhancement. | Reduction in the lesion size and enhancement. | Reduction in the lesion size and enhancement. | Notable reduction in gadolinium enhancing foci and no evidence of new lesion. | The lesion decreased in size, but a new lesion was detected. | - | There was no significant change in the lesion after RTX treatments, but after discontinuation of RTX, microhaemorrhages of the lesion increased. | Lesion enhancement disappeared but hydrocephalus and low signal on SWI of the brain surface of the left hemisphere was detected. |
| EDSS at RTX initiation | 1.5 | 2.0 | 6.5 | 3.0 | 6.5 | 7.0 | 9.5 | 9.5 |
| EDSS at last follow-up | 0.0 | 1.0 | 3.0 | 2.0 | 6.5 | Dead (Pulmonary embolism possible) | 9.5 | 9.0 |
| mRS at RTX initiation | 1 | 1 | 4 | 2 | 4 | 4 | 5 | 5 |
| mRS at last follow-up | 0 | 1 | 4 | 2 | 4 | Dead (Pulmonary embolism possible) | 5 | 4 |
| Follow-up duration (time after starting RTX) | 18m | 15m | 17m | 40m | 6m | 10d | 36m | 38m |
| RTX adverse effects | Pneumonia | - | - | Tuberculosis | - | - | - | - |

RTX, rituximab; IVMP, intravenous methylprednisolone; PDN, prednisone; CYC, cyclophosphamide; MMF, mycophenolate mofeti; CsA, cyclosporine A; MRI, magnetic resonance imaging; EDSS, Expanded Disability Status Scale; mRS, modified Rankin score
